# Real-World Outcomes and Prognostic Factors of Polymyxin B Hemoperfusion in Severe Sepsis and Septic Shock: A Seven-Year Single-Center Cohort Study from Taiwan

**DOI:** 10.3390/life15081317

**Published:** 2025-08-20

**Authors:** Wei-Hung Chang, Ting-Yu Hu, Li-Kuo Kuo

**Affiliations:** 1 Department of Critical Care Medicine, MacKay Memorial Hospital, Taipei 10449, Taiwan; peacejaycool@gmail.com (W.-H.C.); lmn4093@gmail.com (L.-K.K.); 2 Department of Medicine, Mackay Medical College, New Taipei City 25245, Taiwan

**Keywords:** polymyxin B hemoperfusion, severe sepsis, septic shock, intensive care unit, endotoxemia, APACHE II score, continuous renal replacement therapy, real-world data, prognostic factors

## Abstract

**Background**: Severe sepsis and septic shock remain major contributors to ICU mortality. Polymyxin B hemoperfusion (PMX-HP) has been widely adopted as adjunctive therapy in Asian ICUs for endotoxemia, but its real-world effectiveness and prognostic factors remain uncertain, especially in high Gram-negative settings. **Methods**: This retrospective cohort study included 64 adult patients with severe sepsis or septic shock who received at least one session of PMX-HP in a 25-bed tertiary medical ICU in Taiwan between July 2013 and December 2019. Demographic, clinical, microbiological, and treatment data were extracted. The primary outcome was 28-day mortality. Prognostic factors were analyzed using logistic regression. **Results**: The mean age was 66.1 ± 12.3 years; 67.2% were male. Pneumonia (29.7%) and intra-abdominal infection (18.8%) were the most common sources of sepsis, with E. coli and K. pneumoniae as leading pathogens. Median APACHE II score at ICU admission was 26 (IQR 21–32), and 79.7% received two PMX-HP sessions. The 28-day mortality rate was 46.9%, with ICU and hospital mortality both 53.1%. Non-survivors were older, had higher APACHE II scores, and more frequent use of **continuous renal replacement therapy (CRRT). Positive changes in vasoactive-inotropic score (VIS)** after PMX-HP were also more common among non-survivors. Multivariate analysis identified advanced age, higher APACHE II score, and CRRT requirement as independent predictors of mortality. **Conclusions**: In this real-world Asian ICU cohort, PMX-HP was used mainly for severe cases with a high disease burden and Gram-negative predominance. Despite its frequent use, overall mortality remained high. Prognosis was primarily determined by underlying disease severity, organ dysfunction (especially renal failure), and persistent hemodynamic instability. In this high-severity cohort, mortality appeared to be primarily driven by baseline organ dysfunction and persistent hemodynamic instability; PMX-HP session number or sequencing showed no association with survival. Given the absence of a contemporaneous non-PMX-HP control group, mortality observations in this cohort cannot be causally attributed to PMX-HP and should be interpreted with caution as hypothesis-generating rather than definitive evidence of efficacy. Further multicenter studies are needed to clarify the optimal role of PMX-HP in modern sepsis management.

## 1. Introduction

Severe sepsis and septic shock remain major challenges in intensive care units (ICUs) globally, leading to significant morbidity and mortality despite advances in early recognition and standardized management, as highlighted by the Surviving Sepsis Campaign guidelines [1,2,3]. Outcomes remain particularly poor in patients with refractory shock and multi-organ dysfunction, even with aggressive antimicrobial therapy and hemodynamic optimization. During the study period (2013–2019), the term “severe sepsis” was used to describe sepsis with associated organ dysfunction, in accordance with the conventional definitions prior to the adoption of Sepsis-3 in 2016 [3]. Large-scale epidemiological studies have confirmed the persistently high burden of infection and mortality in ICU patients worldwide [4,5].

A central driver of sepsis pathophysiology is endotoxin, or lipopolysaccharide (LPS), primarily produced by Gram-negative bacteria. Endotoxin initiates a cascade of inflammatory signaling through toll-like receptor 4 (TLR4), leading to cytokine storm, endothelial activation, microvascular leakage, and profound vasodilation. These events collectively impair tissue perfusion and precipitate multi-organ failure and death [6]. Recent studies also indicate that endotoxemia may be present in non-Gram-negative sepsis, including fungal and viral infections, possibly via gut barrier dysfunction and bacterial translocation under critical illness [7,8,9].

Polymyxin B hemoperfusion (PMX-HP) is an extracorporeal therapy developed to selectively adsorb circulating endotoxin. By immobilizing polymyxin B on polystyrene fibers, the PMX-HP cartridge facilitates endotoxin removal without the nephrotoxicity and neurotoxicity associated with systemic polymyxin B use. Since its introduction, PMX-HP has been widely utilized across Asia, especially in Japan and Taiwan, where Gram-negative sepsis predominates. Multiple early clinical studies and real-world reports have suggested improved hemodynamics and reduced vasopressor needs in septic shock patients, stimulating its broader adoption [10,11,12,13,14,15,16].

However, the efficacy of PMX-HP remains controversial on the international stage. While some meta-analyses and randomized controlled trials (RCTs) have indicated mortality benefits in highly selected patient subgroups, larger Western RCTs have failed to show clear survival advantages over standard care in broader populations [17,18,19,20]. These discrepancies may stem from heterogeneity in patient selection, timing of intervention, disease severity, and significant regional differences in microbiological epidemiology and clinical practice [21,22,23].

Notably, Asia—including Taiwan—faces a uniquely high burden of Gram-negative sepsis, with local epidemiology and resource allocation differing substantially from Western countries. PMX-HP is reimbursed by Taiwan’s National Health Insurance (NHI) for refractory septic shock, but standardized protocols and evidence-based selection criteria are lacking, leading to potential variation in real-world utilization [24,25,26,27,28].

In addition to these knowledge gaps, Taiwan’s unique health policy landscape presents both opportunities and challenges for the implementation of advanced sepsis therapies. The National Health Insurance (NHI) system, which provides nearly universal coverage, currently reimburses PMX-HP for refractory septic shock; however, the absence of uniform national protocols or explicit biomarker-based selection criteria has led to significant heterogeneity in clinical practice [29,30,31,32]. As a result, indications for PMX-HP often rely heavily on physician experience and resource availability, rather than standardized guidelines or real-time risk stratification tools. This pragmatic approach may promote access to advanced therapy for critically ill patients, but also raises questions of cost-effectiveness, equitable resource allocation, and optimal patient selection.

Resource allocation in Taiwanese ICUs is further complicated by high patient volumes, evolving patterns of antimicrobial resistance, and the need to balance state-of-the-art interventions with sustainability and system-wide efficiency [33,34,35]. With the growing burden of multi-drug-resistant Gram-negative infections and the rising costs of critical care, health policymakers are increasingly called upon to develop evidence-based frameworks that integrate epidemiological realities, health economics, and clinical outcomes [36,37].

Moreover, there is a paucity of high-quality, real-world evidence focusing on East Asian populations, who may differ significantly from Western cohorts in genetic background, comorbidities, health system organization, and cultural factors influencing care delivery. These differences extend beyond microbiological epidemiology to include variable rates of chronic diseases, patterns of ICU admission and resource utilization, and even patient and family expectations regarding end-of-life care and aggressive interventions [38,39]. Existing global guidelines, largely derived from Western RCTs, may not fully capture the complexity of the East Asian context, underscoring the urgent need for region-specific research and locally adapted practice standards [2,40].

Against this backdrop, our study is uniquely positioned to provide contextually relevant data from Taiwan, bridging the knowledge gap between global evidence and local clinical realities. The strengths of our study are threefold: (1) it offers robust, real-world clinical data from a Taiwanese tertiary ICU—a high-volume center facing a typical East Asian epidemiological profile; (2) it incorporates detailed clinical, microbiological, and hemodynamic variables, allowing granular assessment of patient characteristics and risk factors; and (3) it is uniquely positioned to inform evidence-based patient selection and resource optimization for PMX-HP in Asian ICUs, bridging the gap between global trial evidence and local clinical realities.

In this retrospective cohort study, we evaluated the clinical characteristics, treatment details, and outcomes of patients with severe sepsis and septic shock treated with PMX-HP in a Taiwanese tertiary ICU. We further identified key prognostic factors associated with mortality, providing new real-world insights to guide future clinical decision-making, policy formulation, and the design of prospective, regionally relevant sepsis trials.

In Japan and selected research settings, endotoxin activity assay (EAA) has been utilized as a quantitative biomarker to guide early initiation of PMX-HP, enabling more precise patient selection and potential survival benefit.

## 2. Materials and Methods

### 2.1. Study Design and Setting

This retrospective cohort study was conducted in the medical intensive care unit (ICU) of a tertiary medical center in Taiwan. The ICU comprises 25 beds and provides advanced care for critically ill adult patients. The study period spanned from 1 July 2013, to 31 December 2019.

### 2.2. Patient Population

All adult patients (aged ≥ 20 years) diagnosed with **severe sepsis** (defined as sepsis-induced organ dysfunction, according to the pre-Sepsis-3 definitions used during the study period) or septic shock, as per the Surviving Sepsis Campaign: International Guidelines for Management of Sepsis and Septic Shock (2016), who received at least one session of polymyxin B hemoperfusion (PMX-HP) during their ICU stay, were eligible for inclusion.

Exclusion criteria were as follows:

- Age < 20 years.
- Pregnancy.
- Participation in another clinical trial within one month.
- Receipt of organ transplantation within one year.
- Anticipated survival < 30 days (e.g., advanced malignancy as assessed by the attending physician).
- Cardiopulmonary resuscitation within four weeks prior to ICU admission.
- Do-not-resuscitate (DNR) order or palliative care decision at admission.
- Diagnosed HIV infection.
- Active uncontrolled bleeding within 24 h.
- Brain death at admission.
- Child–Pugh C liver failure.
- Use of blood purification therapy (CVVH, hemodialysis, hemofiltration, plasma exchange) within 24 h prior to enrollment.
- Hemophilia.
- History of allergy to polymyxin B, heparin, or extracorporeal circuit materials.

### 2.3. Patient Flow

**Figure 1:**
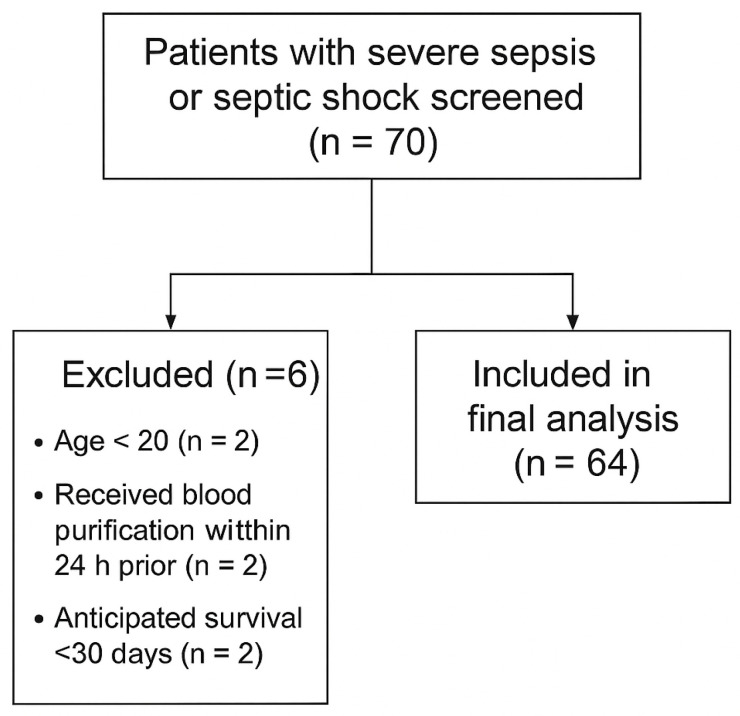
**Patient enrollment and exclusion flow diagram.** *Legend*: Flow diagram showing the number of patients screened (*n* = 70), excluded (*n* = 6; age < 20 [*n* = 2], received blood purification within 24 h prior [*n* = 2], anticipated survival < 30 days [*n* = 2]), and included in the final analysis (*n* = 64).

A total of 70 patients with severe sepsis or septic shock were initially screened during the study period. After applying inclusion and exclusion criteria, 64 patients who received at least one session of polymyxin B hemoperfusion (PMX-HP) were enrolled in the final analysis (see Figure 1: Patient enrollment and exclusion flow diagram).

### 2.4. Data Collection

Clinical, laboratory, and microbiological data were retrospectively extracted from electronic medical records using a standardized data collection form. Collected variables included age, sex, body weight, underlying comorbidities, source of sepsis, causative pathogens, APACHE II score at ICU admission, details of PMX-HP treatment (number of sessions, session duration, sequential versus non-sequential therapy), and use of adjunctive organ support (continuous renal replacement therapy [CRRT], extracorporeal membrane oxygenation [ECMO]).

APACHE II scores were calculated based on the worst values within the first 24 h of ICU admission. The vasoactive-inotropic score (VIS) was computed as follows:

VIS = dopamine dose (μg/kg/min) + dobutamine dose (μg/kg/min) + 100 × epinephrine dose (μg/kg/min) + 10 × milrinone dose (μg/kg/min) + 10,000 × vasopressin dose (U/kg/min) + 100 × norepinephrine dose (μg/kg/min).

+ A “positive change” in VIS was defined as an absolute increase in the total VIS from pre-PMX-HP (T2) to 24 h after the final PMX-HP session (T3), calculated as T3 − T2 > 0, indicating worsening or persistent hemodynamic instability.

+ A “negative change” was defined as T3 − T2 < 0, reflecting improvement in hemodynamic status.

+ A value of zero indicated no net change.

For the purpose of dynamic assessment, T2 was defined as the VIS score measured immediately prior to the first PMX-HP session, and T3 as the VIS score recorded approximately 24 h after the final PMX-HP session.

All ICU staff adhered to standard airborne precautions, including N95 respirators during aerosol-generating procedures (e.g., intubation, bronchoscopy), and all patient care areas complied with hospital infection control protocols. No occupational transmission of tuberculosis or multi-drug-resistant organisms was reported during the study period.

### 2.5. PMX-HP Intervention

Polymyxin B hemoperfusion was performed according to institutional ICU protocol using a Toraymyxin^®^ cartridge (Toray Industries, Tokyo, Japan). Each session was typically prescribed for 2–6 h at a blood flow rate determined by the attending intensivist. The total number of sessions, session duration, and the decision for sequential therapy were based on clinical judgment and patient response.

### 2.6. Organ Support Protocols

CRRT was initiated according to institutional protocols in patients with oliguric or anuric renal failure, severe metabolic acidosis, or refractory volume overload unresponsive to conservative measures. Extracorporeal membrane oxygenation (ECMO) was considered for refractory hypoxemia or circulatory collapse despite maximal conventional support and after multidisciplinary assessment.

### 2.7. Outcome Measures

Primary outcome:

- All-cause 28-day mortality after PMX-HP initiation.

Secondary outcomes:

- ICU and hospital mortality.
- ICU and hospital length of stay.
- Requirement and timing of CRRT and extracorporeal membrane oxygenation (**ECMO**).
- Changes in vasoactive-inotropic score (VIS) before (T2) and 24 h after (T3) PMX-HP.
- Additional clinical endpoints as per protocol (e.g., ventilator-free days, laboratory trends).

### 2.8. Statistical Analysis

Continuous variables were expressed as mean ± standard deviation (SD) or median (interquartile range (IQR)), according to their distribution (normality assessed via the Kolmogorov–Smirnov test). Categorical variables were summarized as counts and percentages. Comparisons between survivors and non-survivors were performed using the Student’s *t*-test or Mann–Whitney U test for continuous variables and the chi-square or Fisher’s exact test for categorical variables, as appropriate.

Prognostic factors for mortality were assessed using univariate and multivariate logistic regression analyses, including variables with clinical relevance or statistical significance (*p* < 0.10 in univariate analysis) in the final model. Odds ratios (ORs) and 95% confidence intervals (CIs) were calculated. Statistical significance was set at *p* < 0.05. All analyses were performed using IBM SPSS Statistics version 26.0 (IBM Corp., Armonk, NY, USA).

### 2.9. Missing Data Handling

Missing data were handled using pairwise deletion, in accordance with previously published ICU sepsis studies [Payen et al., Intensive Care Med. 2015;41(6):975–984]. Cases with unavailable laboratory or hemodynamic values at critical time points (e.g., within 24–72 h post-PMX-HP) were not imputed and were reported as missing.

### 2.10. Ethics Statement

This study was approved by the Institutional Review Board of MacKay Memorial Hospital (IRB No. 18MMHIS198e) and conducted in accordance with the Declaration of Helsinki and institutional guidelines. Due to the retrospective and anonymized nature of the analysis, the requirement for informed consent was waived.

## 3. Results

### 3.1. Patient Flow

A total of 70 patients with severe sepsis or septic shock were screened for eligibility between July 2013 and December 2019. Six patients were excluded due to age < 20 years (*n* = 2), receipt of blood purification within 24 h prior to enrollment (*n* = 2), or anticipated survival < 30 days (*n* = 2). Sixty-four patients received at least one session of PMX-HP and were included in the final analysis (Figure 1).

### 3.2. Baseline Characteristics

**Figure 2:**
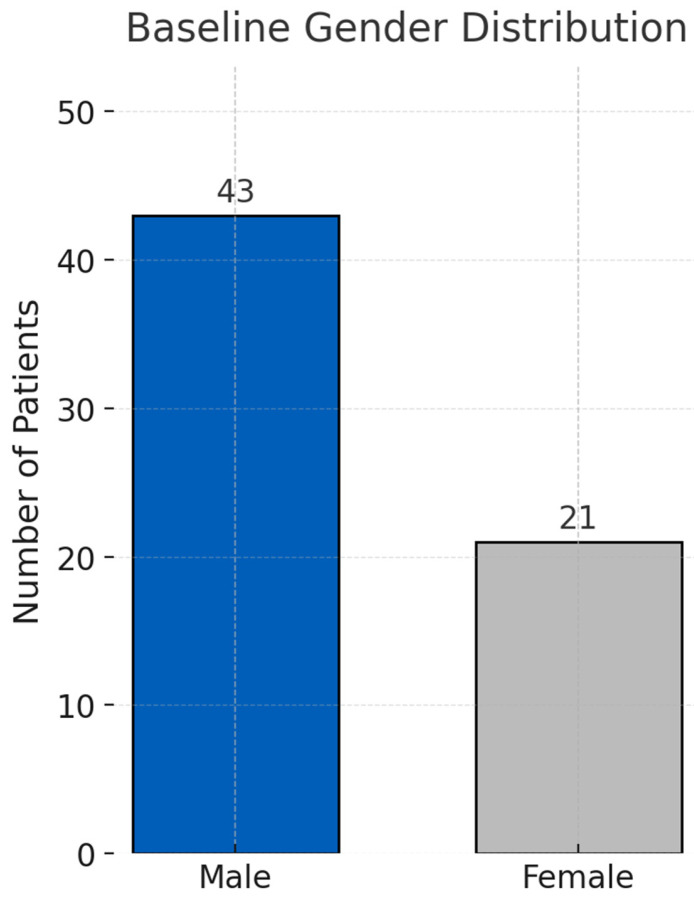
**Baseline gender distribution in the study cohort.** *Legend*: Bar chart illustrating the number and percentage of male and female patients who received PMX-HP for severe sepsis or septic shock in the ICU between July 2013 and December 2019.

Among the 64 patients analyzed, the mean age was 66.1 ± 12.3 years (range, 37–94), and 43 (67.2%) were male (Figure 2). The median body weight was 66.5 kg (IQR, 57.3–72.9 kg). The median APACHE II score at ICU admission was 26 (IQR, 21–32); 95.3% had APACHE II > 12.

**Figure 3:**
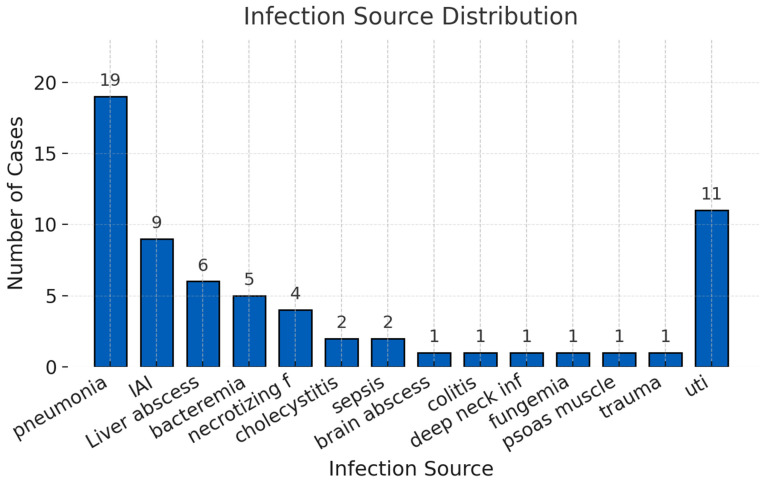
**Distribution of infection sources among patients treated with PMX-HP.** *Legend*: Bar chart showing the frequency and distribution of primary infection sources in the patient cohort.

**Figure 4:**
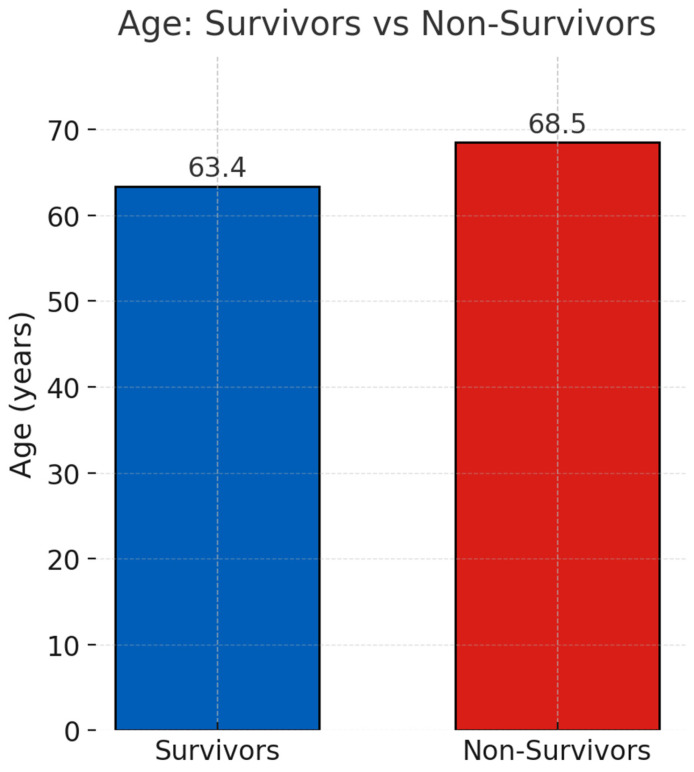
**Age distribution of survivors versus non-survivors.** *Legend*: Bar chart comparing the mean age between survivors and non-survivors after PMX-HP therapy.

The most frequent sources of sepsis were pneumonia (*n* = 19, 29.7%), intra-abdominal infection (*n* = 12, 18.8%), urosepsis (*n* = 11, 17.2%), skin and soft tissue infection (*n* = 6, 9.4%), and liver abscess (*n* = 6, 9.4%) (Figure 3). Other sources included bacteremia (*n* = 5), brain abscess (*n* = 1), trauma (*n* = 1), and fungemia (*n* = 1). **Primary infection sources are shown in Figure 2; organism distribution is summarized in Figure 4.**

The leading pathogens were *Escherichia coli* (*n* = 20, 31.3%), *Klebsiella pneumoniae* (*n* = 14, 21.9%), *Staphylococcus aureus* (*n* = 6, 9.4%), and, less commonly, *A. baumannii*, *S. maltophilia*, *Aspergillus*, and *Candida* spp. The distribution and frequency of pathogens are shown in Figure 4.

The majority of patients (79.7%) received two sessions of PMX-HP; 14.1% received one session, 3.1% received three sessions, and 3.1% received four sessions. Session duration was predominantly 2 h (87.5%). Sequential therapy was used in 51.6% of cases (Table 1).

### 3.3. Microbiological Resistance and Antibiotic Usage

Among the 64 patients, multidrug-resistant (MDR) Gram-negative organisms were identified in 17 cases (26.6%), including extended-spectrum β-lactamase (ESBL)-producing Escherichia coli (*n* = 7), carbapenem-resistant Klebsiella pneumoniae (CRKP, *n* = 6), and multidrug-resistant Acinetobacter baumannii (*n* = 4). Methicillin-resistant Staphylococcus aureus (MRSA) was isolated in three patients. No vancomycin-resistant enterococci (VRE) or pan-drug resistant strains were identified, as shown in Figure 4.

Empiric antibiotic regimens most commonly included meropenem (*n* = 44, 68.8%), piperacillin–tazobactam (*n* = 21, 32.8%), and tigecycline (*n* = 9, 14.1%), with subsequent de-escalation or escalation based on culture results. Colistin was administered in seven patients (10.9%), primarily for CRKP or A. baumannii.

A subgroup analysis comparing 28-day mortality between patients with MDR pathogens versus non-MDR pathogens showed no statistically significant difference (52.9% vs. 43.2%, *p* = 0.42). However, patients with MDR infections more frequently required early CRRT (64.7% vs. 38.6%, *p* = 0.048) and had longer ICU stays.

### 3.4. Clinical Outcomes

The 28-day mortality rate was 46.9% (30/64), while both ICU and hospital mortality rates were 53.1% (34/64) (Table 2). The median ICU length of stay was 9.3 days (IQR, 4.4–21.1), and the median hospital length of stay was 20.5 days (IQR, 8–34.6). Median duration of mechanical ventilation was not consistently recorded and was thus excluded from analysis. We did not impute missing ventilator durations to avoid bias from irregular documentation.

Continuous renal replacement therapy (CRRT) was initiated within 24 h after shock in 45.3% of patients and within 28 days in 67.2%. ECMO was utilized in 4.7% of cases. These are descriptive outcomes of the entire cohort; no statistical comparisons were performed in this table. Comparative analysis between survivors and non-survivors is presented in Table 3.

### 3.5. Comparison Between Survivors and Non-Survivors

Compared with survivors (*n* = 30), non-survivors (*n* = 34) were older (68.5 ± 9.8 vs. 63.4 ± 14.3 years, *p* = 0.098), had higher APACHE II scores (27.5 ± 6.5 vs. 24.2 ± 6.0, *p* = 0.049), and were more often male (79.4% vs. 53.3%, *p* = 0.03) (Table 3, Figure 4). Baseline age and gender distributions are summarized in Table 1. As their visual representation did not provide additional analytical value, Figure 2 and Figure 4 were removed for conciseness.

Median ICU LOS was 9.3 days (IQR, 7.1–19.9) in survivors vs. 9.5 days (IQR, 3–23) in non-survivors (*p* = 0.7848); hospital LOS was significantly longer in survivors (27.9 days, IQR 18–53.3) than in non-survivors (13 days, IQR 4.5–23, *p* = 0.0074).

CRRT was required in 55.9% of non-survivors vs. 33.3% of survivors within 24 h (OR: 2.53, *p* = 0.0227) and in 82.4% vs. 50.0% within 28 days (OR: 4.67, *p* = 0.0059).

The number of PMX-HP sessions, use of sequential therapy, and session duration did not differ significantly between groups (all *p* > 0.1).

### 3.6. Hemodynamic Response and VIS Score

We prespecified VIS change as T3–T2, with positive values indicating worsening hemodynamic status. A positive change in vasoactive-inotropic score (VIS; T3–T2 > 0) was observed in 35.3% of non-survivors versus 6.7% of survivors (OR: 1.18, *p* = 0.0057), indicating that persistent hemodynamic instability was a predictor of mortality. As defined in the Materials and Methods section, a positive change indicates an absolute increase in VIS from T2 to T3, a negative change indicates a decrease, and zero denotes no change. ECMO was used in two non-survivors (5.9%) and one survivor (3.3%, *p* = 0.63).

### 3.7. Prognostic Factors for Mortality

Univariate analysis (group comparison) showed significant differences in APACHE II scores, CRRT use, and VIS score change between survivors and non-survivors. Variables with *p* < 0.10 were further evaluated in a multivariate logistic regression model. In this model, CRRT within 28 days (adjusted OR: 4.67, *p* = 0.0059), higher APACHE II score (adjusted *p* = 0.0437), and positive VIS score change (adjusted OR: 1.18, *p* = 0.0057) remained independently associated with 28-day mortality (Table 4).

In multivariate analysis, CRRT within 28 days (adjusted OR: 4.67, *p* = 0.0059), higher APACHE II score (adjusted *p* = 0.0437), and positive VIS score change (adjusted OR: 1.18, *p* = 0.0057) remained independently associated with mortality. The number and duration of PMX-HP sessions and use of sequential therapy were not associated with outcome.

### 3.8. Figures and Tables

In summary, mortality in this real-world ICU cohort was primarily driven by underlying disease severity, renal failure requiring CRRT, and persistent hemodynamic instability after PMX-HP. PMX-HP session number, duration, and sequencing were not associated with improved survival. This absence of a contemporaneous control group remains a key limitation and should be addressed in future research.

## 4. Discussion

This retrospective cohort study provides comprehensive, real-world evidence on the clinical use and prognostic impact of polymyxin B hemoperfusion (PMX-HP) for severe sepsis and septic shock in a Taiwanese tertiary ICU. Despite frequent use of PMX-HP, mortality remained high (28-day mortality 46.9%; ICU/hospital mortality 53.1%), consistent with global multicenter studies, highlighting the persistent challenge of managing advanced multi-organ dysfunction in critical illness.

### 4.1. Comparison with International Guidelines, RCTs, and Meta-Analyses

Initial optimism for PMX-HP stemmed from Japanese and European studies (EUPHAS, Toraymyxin registry) showing improved hemodynamics and reduced mortality in highly selected septic shock populations [13,26,29]. However, large RCTs (ABDOMIX, EUPHRATES) failed to demonstrate a consistent survival benefit in unselected cohorts, emphasizing the necessity for stratification by endotoxin activity, disease severity, and early intervention [7,17,30]. Recent meta-analyses—including those by Chang et al., Fujii et al., and Suzuki et al.—further support the notion that any mortality benefit is limited to high-risk subgroups with early therapy and elevated endotoxin levels [10,11,20,24].

Our findings support these observations: disease severity, organ failure (notably renal dysfunction), and persistent hemodynamic instability are the strongest prognostic determinants, regardless of adjunctive therapy. The number or sequencing of PMX-HP sessions did not influence survival, aligning with RCT and registry data. Similarly, session duration was not significantly associated with survival outcomes in our cohort (*p* > 0.1).

### 4.2. Asian and Taiwanese Clinical Reality

Unlike Western ICUs, Taiwan and much of East Asia remain dominated by Gram-negative sepsis, especially *E. coli* and *K. pneumoniae*—a fact confirmed by our cohort. This epidemiology shapes PMX-HP practice: in Taiwan, PMX-HP is typically reserved for the most critically ill, vasopressor-dependent patients. Compared with Japan, where national guidelines mandate biomarker-driven early intervention (e.g., endotoxin activity assay (EAA)), Taiwan’s National Health Insurance (NHI) policy is less standardized, with clinical decisions often relying on APACHE II, infection source, and organ failure rather than strict biomarker guidance [25,39]. This pragmatic approach, though reflecting real-world resource allocation, can lead to both overuse and underuse, potentially diluting PMX-HP’s benefits in the absence of rigorous patient selection.

### 4.3. Subgroup and Real-World Stratification

Our results align with recent Asian registry studies (e.g., Matsuda et al.) showing that only early intervention, severe Gram-negative infection, and preserved baseline organ function predict better outcomes [24]. Meta-analyses also suggest that only patients with high endotoxemia and refractory shock may benefit, while broader inclusion dilutes survival signals [11,20]. The lack of routine EAA testing in our center means PMX-HP is mostly reserved for high-severity cases, which differs from many Western RCT populations.

### 4.4. Health Economics and Policy Implications

Although Taiwan’s NHI reimburses PMX-HP for refractory septic shock, payment criteria are not as stringent as in Japan, leading to potential inefficiencies and disparities. Our data suggest that future policy should incorporate dynamic risk markers—such as VIS, renal failure status (early/late CRRT), and validated severity scores (APACHE II)—to guide therapy and optimize resource use. Broader adoption of biomarker-driven protocols and real-time data integration may further improve both cost-effectiveness and outcomes. Absent routine EAA testing, clinicians likely initiated PMX-HP based on global severity and shock physiology rather than endotoxemia burden, which may have diluted any treatment effect by including patients less likely to benefit.

### 4.5. Prognostic Indicators and Practical Recommendations

A persistently elevated or increasing VIS score after PMX-HP was a robust, independent predictor of mortality, confirming global reports that hemodynamic instability is central to outcome in sepsis. Both early and late CRRT requirements also strongly predicted poor prognosis, underscoring the need for multidisciplinary, integrated critical care that goes beyond single-modality interventions.

### 4.6. Study Limitations

This study is limited by its retrospective, single-center design, lack of a non-PMX-HP control group, and absence of routine EAA testing. Some data were missing due to real-world clinical constraints but were handled using validated pairwise deletion. We acknowledge that the retrospective selection process and the application of specific exclusion criteria, such as anticipated survival < 30 days or recent organ transplantation, may have introduced selection bias. This could result in the exclusion of patients with more severe illness or limited life expectancy, potentially yielding a study cohort with a relatively lower baseline risk profile than the general ICU sepsis population. While these criteria aimed to create a more homogeneous group for meaningful analysis, this inherent limitation must be considered when interpreting the external validity and generalizability of our findings.

Second, as a non-randomized, observational study, the number and timing of PMX-HP sessions were determined solely by clinician judgment rather than by protocol or randomization. Consequently, potential confounding by indication cannot be excluded: patients with greater severity of illness or refractory shock may have been preferentially selected to receive additional or earlier sessions, while other key confounders—including baseline endotoxin activity, detailed comorbidities, and prior antibiotic exposure—were not systematically collected or controlled for in the analysis. Additionally, unmeasured variables such as frailty, socioeconomic status, or pre-ICU care may also have influenced both treatment allocation and outcomes. This limits the internal validity and precludes causal inference regarding the effect of PMX-HP on mortality.

Third, data were retrospectively extracted from electronic health records, which introduces the potential for information and measurement bias. Both exposures (such as accurate calculation of VIS, precise session timing/duration) and outcomes (including the cause of death and secondary endpoints) may have been misclassified due to inconsistent or incomplete clinical documentation. Furthermore, the absence of standardized protocols for recording PMX-HP session details and sequential therapy could have led to additional variability in treatment delivery and data collection. While we attempted to mitigate these risks through the cross-validation of key variables and careful chart review, some degree of measurement inconsistency remains unavoidable and may have influenced the robustness of our findings.

Fourth, the extended study period (2013–2019) encompassed substantial changes in ICU practice, sepsis management protocols, and antimicrobial stewardship, which may have influenced both the clinical decision-making process and patient outcomes (temporal bias). Over the course of the study, updates to sepsis bundles, changes in empiric antibiotic strategies, and resource allocation shifts could have contributed to unmeasured confounding by time. Immortal time bias is also possible, as patients had to survive long enough in the ICU to receive subsequent PMX-HP sessions, potentially inflating the apparent benefit in multi-session groups. Similarly, lead-time bias may have been introduced if the timing of PMX-HP initiation was not standardized or analyzed relative to the onset of sepsis or shock. While we report our findings in the context of evolving real-world care, these sources of temporal bias limit the comparability across different time periods and may affect the interpretation of our results.

Fifth, missing data were handled using pairwise deletion, consistent with established ICU sepsis cohort methodology. However, this approach assumes that data are missing completely at random. If missing laboratory or hemodynamic data were more likely to occur in patients with higher illness severity or adverse outcomes, this could have introduced bias by disproportionately excluding more severe cases from certain analyses, thus affecting effect size estimation and internal validity. We acknowledge this potential source of bias and recognize that more sophisticated imputation strategies (e.g., multiple imputation, maximum likelihood estimation) could help address this limitation in future studies.

Sixth, the absence of a contemporaneous control group—such as patients with severe sepsis or septic shock who did not receive PMX-HP—precludes definitive attribution of clinical outcomes to hemoperfusion therapy rather than to standard ICU care or natural disease progression. While we utilized multivariate regression to adjust for available confounders, causal inference is fundamentally limited by the study design. Therefore, the associations observed in this study should be interpreted as hypothesis-generating rather than providing definitive evidence of PMX-HP efficacy. Future prospective studies with appropriate control groups are warranted to establish the causal effects of PMX-HP in this population.

Seventh, adverse events potentially attributable to PMX-HP—such as thrombocytopenia, hypersensitivity/anaphylaxis, hypotension, and circuit-related complications—were not systematically captured or adjudicated in our retrospective dataset. As a result, we cannot reliably quantify the incidence, severity, or risk profile of these adverse effects, nor directly compare them with background rates in standard ICU care. This limitation precludes a comprehensive risk–benefit assessment of PMX-HP and may have resulted in under-recognition of clinically significant safety signals. Future prospective studies with standardized adverse event monitoring and reporting are needed to better inform clinical risk stratification and guideline recommendations for PMX-HP use.

Despite these limitations, our findings accurately reflect contemporary ICU practice in Taiwan and greater Asia, and thus have high relevance for regional policy and clinical strategy.

### 4.7. Future Directions

Future research should focus on multicenter, prospective studies with biomarker-guided patient selection, standardized PMX-HP and CRRT protocols, and robust cost-effectiveness analyses. Future studies should also incorporate robust missing data handling techniques to reduce bias. Greater collaboration across Asia could enable the development of evidence-based, regionally adapted guidelines for advanced sepsis therapies, making the region a leader in precision critical care. Our findings suggest that, although multidrug-resistant (MDR) infections were prevalent, their direct association with mortality was less pronounced compared with factors such as organ failure and VIS scores. This aligns with prior literature indicating that the host response may play a more decisive prognostic role than pathogen resistance alone. To address these limitations, future prospective studies should include well-matched control groups to allow more definitive causal inferences regarding PMX-HP efficacy. In light of the persistent high mortality, given these limitations, real-world PMX-HP use should be restricted to those with highest potential benefit and ideally within prospective registries or trials.

## 5. Conclusions

In this real-world cohort of critically ill patients with severe sepsis and septic shock treated with PMX-HP in a Taiwanese tertiary ICU, mortality remained high despite advanced supportive care. Prognosis was determined primarily by underlying disease severity, renal failure, and persistent hemodynamic instability, rather than the number or sequencing of PMX-HP sessions. Similarly, session duration and use of sequential hemoperfusion showed no significant association with survival, further underscoring the dominance of disease severity and organ dysfunction in prognostic outcomes. Importantly, the absence of a contemporaneous control group precludes definitive attribution of clinical outcomes to PMX-HP; therefore, the observed associations should be considered hypothesis-generating rather than definitive evidence of efficacy. These findings highlight the urgent need for careful patient selection, dynamic risk stratification (using tools such as APACHE II and VIS scores), and the integration of early renal and hemodynamic markers into PMX-HP decision-making.

Given Taiwan’s and Asia’s unique epidemiological and policy context, future multicenter, biomarker-driven research is needed to clarify optimal PMX-HP indications, improve cost-effectiveness, and ensure equitable resource allocation. Policymakers and clinicians should strive to develop and adopt regionally adapted, evidence-based criteria for blood purification therapies in sepsis, moving toward precise, multidisciplinary, and outcome-oriented critical care. **Overall, the absence of a contemporaneous control group remains a key limitation; therefore, our findings should be considered hypothesis-generating rather than confirmatory, and future research should address this gap.**

## Figures and Tables

**Table 1:** **Baseline characteristics of patients treated with polymyxin B hemoperfusion (PMX-HP) (*n* = 64).** *Legend*: Demographic, clinical, and infection-related characteristics of all patients with severe sepsis or septic shock who received PMX-HP. Data are presented as mean ± standard deviation (SD), median (interquartile range, IQR), or number (%), as appropriate. Abbreviations: APACHE II, Acute Physiology and Chronic Health Evaluation II; IQR, interquartile range; PMX-HP, polymyxin B hemoperfusion.

Characteristics	Total (*n* = 64)	*p* Value	Min, Max, and IQR
**Age, years, mean (SD)**	66.1 ± 12.3		max 94, min 37, IQR 59–76
**Gender**		0.294	
Male	43 (67.2%)		
Female	21 (32.8%)		
**Body weight, kg, mean (SD)**	66.5 ± 15.2		max 119.7, min 42, IQR 57.3–72.9
**APACHE II score**		<0.0001	
APACHE II at ICU admission, median (IQR)	26 (21–32)		max 38, min 11
APACHE II ≥ 12, ICU admission	3 (4.7%)		
APACHE II < 12, ICU admission	61 (95.3%)		
**Number of PMX-HP sessions**		<0.0001	
1 session	9 (14.1%)		
2 sessions	51 (79.7%)		
3 sessions	2 (3.1%)		
4 sessions	2 (3.1%)		
**PMX used ≥ 2 sessions**		0.0012	

**Table 2:** **Clinical outcomes of patients receiving PMX-HP (*n* = 64).** *Legend*: Key clinical outcomes among all patients who underwent PMX-HP, including ICU and hospital mortality, 28-day mortality, length of stay, and requirement for organ support. Data are presented as median (IQR), mean ± SD, or number (%), as appropriate. Note: This table presents descriptive statistics for the entire cohort without statistical comparison.

Outcomes	Total (*n* = 64)	*p* Value	Mean ± SD
ICU length of stay, median (IQR), days	9.3 (4.4–21.1)		16 ± 18.6
Hospital length of stay, median (IQR), days	20.5 (8–34.6)		28 ± 29.2
Hospital mortality, *n* (%)	34 (53.1%)	0.6171	
ICU mortality, *n* (%)	34 (53.1%)	0.6171	
28-day mortality after PMX-HP, *n* (%)	30 (46.9%)	0.6171	
CRRT within 24 h after shock, *n* (%)	29 (45.3%)	0.0783	
CRRT within 28 d after shock, *n* (%)	43 (67.2%)	0.006	
ECMO use, *n* (%)	3 (4.7%)	<0.0001	

Abbreviations: ICU, intensive care unit; CRRT, continuous renal replacement therapy; ECMO, extracorporeal membrane oxygenation; PMX-HP, polymyxin B hemoperfusion; IQR, interquartile range; SD, standard deviation.

**Table 3:** **Baseline clinical and treatment characteristics by survival status.** *Legend*: Comparison of demographic, clinical, and treatment variables between survivors and non-survivors in the PMX-HP cohort. Data are expressed as mean ± SD, median (IQR), or number (%), as appropriate. Abbreviations: CRRT, continuous renal replacement therapy; APACHE II, Acute Physiology and Chronic Health Evaluation II; IQR, interquartile range; PMX-HP, polymyxin B hemoperfusion; SD, standard deviation.

Characteristics	Survivors (*n* = 30)	Non-Survivors (*n* = 34)	*p* Value 1	*p* Value 2	Min, Max, IQR	Normality	Test 1	Test 2
Age, years, mean (SD)	63.4 ± 14.3	68.5 ± 9.8	0.098			yes	logistic	*t*-test
Gender			0.0299	0.0266	n/s	n/a	logistic	chi-square
Male, *n* (%)	16 (53.3%)	27 (79.4%)				n/a	logistic	chi-square
Female, *n* (%)	14 (46.7%)	7 (29.6%)				n/a	logistic	chi-square
Body weight, kg, median (IQR)	61.35 (51.5–70)	68.35 (54.3–74.5)		0.5036		no	logistic	Mann-Whitney U
APACHE II at ICU admission, mean (SD)	24.2 ± 6	27.5 ± 6.5	0.0489	0.0437		yes	logistic	*t*-test
APACHE II ≥ 12, ICU admission, *n* (%)	29 (96.7%)	32 (94.1%)						
APACHE II < 12, ICU admission, *n* (%)	1 (3.3%)	2 (5.9%)						
Number of PMX-HP sessions, *n* (%)			0.0653	0.0243		n/a	logistic	chi-square
1 session	1 (3.3%)	8 (23.5%)						
2 sessions	27 (90%)	24 (70.6%)						
3 sessions	0 (0%)	2 (5.9%)						
4 sessions	2 (6.7%)	0 (0%)						
PMX used ≥ 2 sessions, *n* (%)			0.4412	0.4402		n/a		chi-square

**Table 4:** **Outcomes and risk factors by survival status.** *Legend*: Comparison of key clinical outcomes and risk factors between survivors and non-survivors among patients with severe sepsis or septic shock treated with PMX-HP. Data are presented as median (IQR) or number (%), as appropriate. *p* value1 calculated using Student’s *t*-test or Mann–Whitney U test for continuous variables and chi-square test for categorical variables; *p* value derived from logistic regression. Odds ratios (ORs) are from univariate logistic regression. Abbreviations: IQR, interquartile range; OR, odds ratio; CRRT, continuous renal replacement therapy; ECMO, extracorporeal membrane oxygenation; VIS, vasoactive-inotropic score; PMX-HP, polymyxin B hemoperfusion.

Outcome/Variable	Survivors (*n* = 30)	Non-Survivors (*n* = 34)	*p* Value 1	*p* Value 2	OR (Univariate)
ICU length of stay, median (IQR), days	9.3 (7.1–19.9)	9.5 (3–23)	0.7848	0.7887	
Hospital length of stay, median (IQR), days	27.9 (18–53.3)	13 (4.5–23)	0.0074	0.0009	
CRRT within 24 h after shock, *n* (%)	10 (33.3%)	19 (55.9%)	0.0733	0.0227	2.533
CRRT within 28 d after shock, *n* (%)	15 (50.0%)	28 (82.4%)	0.0078	0.0059	4.667
ECMO use, *n* (%)	1 (3.3%)	2 (5.9%)	0.6346	0.6302	1.812
VIS score change T3–T2 > 0, *n* (%)	2 (6.7%)	12 (35.3%)	0.0035	0.0057	1.177

## Data Availability

The datasets generated and analyzed during the current study are available from the corresponding author on reasonable request. All relevant clinical data are presented within the article.
