# Correction: Chang et al. Real-World Outcomes and Prognostic Factors of Polymyxin B Hemoperfusion in Severe Sepsis and Septic Shock: A Seven-Year Single-Center Cohort Study from Taiwan. *Life* 2025, *15*, 1317

**DOI:** 10.3390/life15121798

**Published:** 2025-11-24

**Authors:** Wei-Hung Chang, Ting-Yu Hu, Li-Kuo Kuo

**Affiliations:** 1 Department of Critical Care Medicine, MacKay Memorial Hospital, Taipei 10449, Taiwan; peacejaycool@gmail.com (W.-H.C.); lmn4093@gmail.com (L.-K.K.); 2 Department of Medicine, Mackay Medical College, New Taipei City 25245, Taiwan

## Error in Authorship

In the original publication [1], the corresponding author was mistakenly indicated as Dr. Wei-Hung Chang. The correct corresponding author is Dr. Ting-Yu Hu. The authors state that the scientific conclusions are unaffected. This correction was approved by the Academic Editor. The original publication has also been updated.
